# Colchicine for prevention of post-operative atrial fibrillation: Meta-analysis of randomized controlled trials

**DOI:** 10.3389/fcvm.2022.1032116

**Published:** 2022-12-01

**Authors:** Peibing Ge, Yu Fu, Qi Su, Mengdi Jin, Li Guo, Congliang Miao, Shun Zhu, Jinqiang Zhuang, Zhi Zhang, Jiang Hong

**Affiliations:** ^1^Shanghai General Hospital of Nanjing Medical University, Shanghai, China; ^2^Department of Cardiology, The Affiliated Huaian No.1 People’s Hospital of Nanjing Medical University, Huaian, Jiangsu, China; ^3^Department of Emergency and Critical Care, Shanghai General Hospital, Shanghai Jiao Tong University School of Medicine, Shanghai, China; ^4^Department of Internal and Emergency Medicine, Shanghai General Hospital, Shanghai Jiao Tong University School of Medicine, Shanghai, China; ^5^Department of Cardiology, Shanghai General Hospital, Shanghai Jiao Tong University School of Medicine, Shanghai, China

**Keywords:** atrial fibrillation, colchicine, post-operative atrial fibrillation, surgery, AF

## Abstract

**Objective:**

This study intended to assess the efficacy of colchicine for prevention of post-operative atrial fibrillation (AF).

**Background:**

Post-operative AF is a common complication of surgery operations. Inflammation plays a crucial role in the pathogenesis of post-operative AF. Colchicine, a potent anti-inflammatory drug, may have a role in mitigating the incidence of post-operative AF.

**Methods:**

We searched Cochrane Library, Web of Science, PubMed, China National Knowledge Infrastructure (CNKI), Database of Chinese sci-tech periodicals (COVIP), and Wanfang Database for randomized controlled trials (RCTs) comparing colchicine versus placebo, or usual care for prevention of post-operative AF. The main outcome was the occurrence of AF post operation, which includes cardiac surgery, lung surgery, or pulmonary vein isolation. The estimated risk ratio (RR) for the occurrence of post-operative AF was evaluated using a random-effects model. The safety end point was the development of any side effects.

**Results:**

A total of 12 RCTs with 2274 patients were eventually included in this meta-analysis, where 1141 patients received colchicine and 1133 patients received placebo or usual care. Perioperative colchicine treatment was related to a decreased incidence of post-operative AF (RR: 0.65; 95% confidence interval [CI]: 0.56 to 0.75, *p*<0.001). Although the incidence of gastrointestinal side effects was increased with colchicine therapy when compared to placebo (RR = 2.49, 95% CI 1.85 to 3.34, *p* < 0.001), the incidence of major adverse events was not increased (RR = 0.86, 95% CI 0.46 to 1.60, *p* = 0.64).

**Conclusion:**

In conclusion, the results of our meta-analysis suggest that colchicine treatment could lower the incidence of post-operative AF. Further studies are needed to determine the optimal colchicine treatment regime to minimize the incidence of adverse events.

## Introduction

Atrial fibrillation (AF) is a prevailing arrhythmia worldwide. It is suggested that AF prevalence is about 3% in adults (1). Post-operative AF is a common complication of surgery operations, especially cardiac surgery. The prevalence of AF after cardiac surgery varies between 10 and 65%, depending on the different surgery type, patient character, and the definition of post-operative AF (2–4). Postoperative AF also occurs in 10- 30% of patients after non-cardiac thoracic surgery (5). Post-operative AF is associated with longer hospital stays, increased incidence of stroke, increased patient mortality, and increased healthcare costs (2, 6, 7). Therefore, prevention of post-operative AF is emphasized by multiple guidelines (1, 8, 9). Many factors may contribute to the development of post-operative AF, such as inflammation, sympathetic/parasympathetic activation, oxidative stress, and structural substrates for electrical re-entry pathways created by surgical procedure (10–12). Prior studies have demonstrated that inflammatory markers like C-reactive protein [CRP] were increased in the group who developed post-operative AF (13–15). Consequently, anti-inflammatory therapy could be useful in preventing the development of post-operative AF. Some anti-inflammation drugs, including corticosteroid therapy, non-steroidal anti-inflammatory drugs (NSAIDs), statin, and colchicine, have been assessed to prevent post-operative AF (2, 16–18).

Colchicine is a potent anti-inflammatory drug which is used in clinic for centuries (19). Unlike NSAIDs and glucocorticosteroids, colchicine has a different mechanism of action that does not relate to the arachidonic acid pathway. Its anti-inflammatory action is mainly related to the inhibition of tubulin polymerization/assembly. Thereby, colchicine inhibits cell mitosis and neutrophil motility (20, 21).

Yet recent studies did not reach an agreement on the efficacy of colchicine in preventing post-operative AF. Some clinical studies and meta analyses favor the use of colchicine to prevent postoperative atrial fibrillation (2, 14, 22–24), while others do not (25–28). In recent years, several new clinical trials on colchicine have been conducted and reported (29–31). In our study, we focus on the available RCTs to evaluate the efficacy and safety of colchicine in prevention of post-operative AF (2, 14, 22, 23, 25–27, 29–33).

## Materials and methods

### Search strategy

This meta-analysis was performed in accordance with the Preferred Reporting Items for Systematic Reviews and Meta-analysis (PRISMA) statement (34). A systematic search was conducted for eligible studies. The following databases were searched: Cochrane Library, Web of Science, PubMed, China National Knowledge Infrastructure (CNKI), Database of Chinese sci-tech periodicals (COVIP), and Wanfang Database. Literature searches were completed on May 1, 2022. The following key words were used: atrial fibrillation, or AF, and colchicine. In order to mitigate publication bias, reference lists within the selected articles and relevant review articles were also scanned for additional studies that may meet inclusion criteria. No language or publication year restriction was applied for the initial extraction of the data. All references of the related trials were reviewed.

### Study selection

Our selection criteria were: (1) only randomized controlled trials (RCTs); (2) study patients who underwent any cardiac surgery, or pulmonary vein isolation (PVI), or any lung surgery; (3) comparisons of colchicine versus placebo, or usual care. Any colchicine treatment regime and dose were accepted. The exclusion criteria were as follows: (1) studies with inadequate outcome data to meet our primary endpoint. Our primary endpoint was the occurrence of post-operative AF, defined as documented AF lasting for more than 30 s following any cardiac surgery, or lung surgery, or PVI. Secondary outcomes of interest were the incidence of adverse events, including adverse effects related to colchicine therapy and major adverse events. Adverse effects related to colchicine therapy referred to gastrointestinal side effects (nausea, diarrhea or vomiting), infection, and hepatotoxicity. Major adverse events were defined as stroke and all-cause death.

**FIGURE 1:**
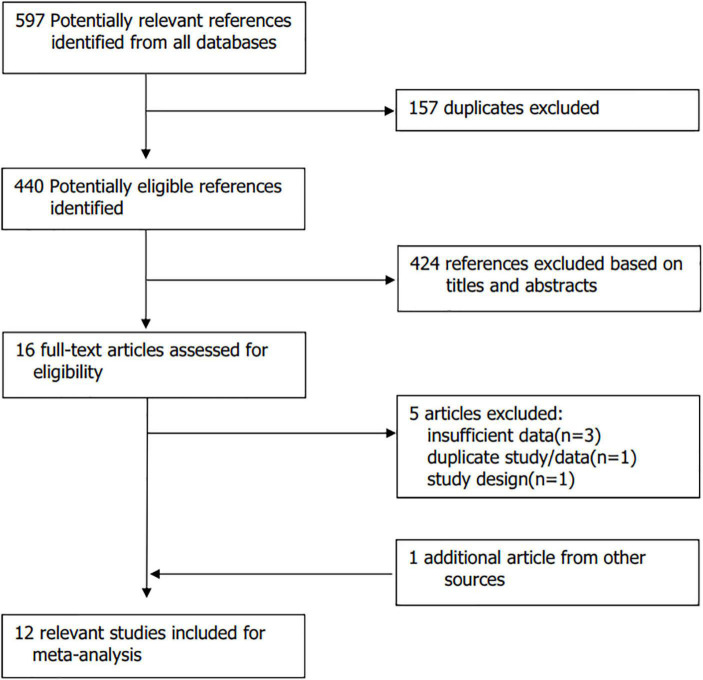
Flow chart. A total of 12 studies were included finally. Reason for exclusion of possibly eligible studies are listed.

Two investigators (Ge Peibing and Fu Yu) independently performed the study selection. During the study selection process, any disagreements were settled by discussion among co-authors. Titles and abstracts of related references were screened for inclusion. Then irrelevant publications were excluded. Full texts of potential articles were downloaded and further evaluated to find whether they meet inclusion criteria (Figure 1). Case reports, editorials, letters, and meta-analyses or systematic reviews were excluded. At last, there were twelve RCTs that evaluated the effectiveness and safety of colchicine in preventing AF after cardiac surgery, or PVI, or lung surgery.

### Quality assessment

The Cochrane Risk of Bias tool by Review Manager were applied to appraise the quality of the included articles. The risks of bias were divided as high risk, or unclear risk, or low risk. Disagreements were resolved by discussion among co-authors.

### Data extraction

We extracted information on publication year, first author’s name, study design, sample size of intervention group and control group, population characteristics, surgery type, dose and time period of colchicine, and outcomes and results. Two independent authors (Ge Peibing and Fu Yu) conducted data extraction. Any disagreements were settled by discussion between the two authors.

### Statistical analysis

Review Manager (RevMan) 5.4 software (available from The Cochrane Collaboration) and STATA 12.0 (StataCorp, College Station, Texas) were applied to statistical analysis. This meta-analysis was performed using the Mantel-Haenszel method and a random effects model. To assess heterogeneity, we applied chi-square test, Tau-square test. To evaluate the proportion of the overall variability in the estimates caused by between-study variation, I-square (I^2^) statistics was performed. I^2^ of <25%, 25% to 50%, and >50% were defined as low, moderate, and high amounts of heterogeneity, respectively. Potential publication bias was evaluated by using the Begg’s test and Egger’s test. To assess additional factors impacting these results, we performed subgroup analysis based on type of surgery. Sensitivity analysis was used by repeating the calculations 12 times and by removing one study at a time.

Continuous data were expressed as mean ± standard deviation (SD). Categorical variables were presented as numbers or percentages. Student’s *t*-test was used for comparison of continuous data of pooled groups of colchicine and placebo participants. Pearson chi-square tests were applied to compare pooled groups of colchicine and placebo participants on categorical variables. These analyses were conducted with the SPSS 22.0 software package (SPSS, Inc., Chicago, IL). All comparisons were 2-sided, and a *p* < 0.05 was considered statistically significant in this meta-analysis.

## Results

### Search results

The study selection process is illustrated in Figure 1. Our initial search yielded 597 references. Of those, a total of 157 references were excluded because of duplicated publication. Of the remaining 440 potentially eligible references, 424 references were removed after screening titles and abstracts because of irrelevance to our topic. The rest 16 references relevant to our topic were selected and the full text of those articles were download for further evaluation. Five publications were excluded because of no available data, duplicate study/data, study design. One additional record identified from relevant review articles was added. Therefore, a total of 12 studies with 1141 patients randomized to receive colchicine and 1133 patients to receive placebo were eventually included in this meta-analysis (2, 14, 22, 23, 25–27, 29–33).

### Baseline characteristics

Table 1 summarizes study baseline characteristics of the 12 included RCTs. Seven RCTs enrolled patients who underwent cardiac surgery [Cardiac surgery included coronary artery bypass grafting (CABG), surgery for aortic diseases, and valve surgery], and 4 RCTs enrolled patients who underwent PVI, and 1 RCT enrolled patients who underwent lung surgery. Colchicine treatment regime varied with respect to dose, initiation time, and treatment duration. Among the included RCTs, 5 trials had 1-month to 3-month treatment duration, and 7 trials had less than 1 month treatment duration. Follow-up ranged from hospital stay time to 15 months. AF was detected either by 12-lead ECG-recording or continuous ECG monitoring.

**TABLE 1:** Characteristics of included studies.

Reference	Study design	Sample size	Participants of colchicine group	Participants of control group	Protocol	Type of surgery	Definition of AF	Follow-up
Bessissow et al. (30)	Randomized controlled study	100	49	51	Colchicine 0.6 mg or placebo starting a few hours before surgery. Postoperatively, patients received colchicine 0.6 mg or placebo twice daily for an additional 9 days	Lung resection surgery	symptomatic AF, or that requires treatment, or that lasts for longer than 30 s.	1 month
Deftereos et al. (23)	A randomized double-blind controlled design	161	81	80	A 3-month course of colchicine 0.5 mg twice daily or placebo.	Pulmonary vein isolation	Symptomatic AF or AF of any duration recorded in electrocardiograms and AF of at least 30 s duration in 48-h Holter	3 months
Deftereos et al. (14)	A randomized, double-blind, controlled trial	206	103	103	Colchicine 0.5 mg twice daily or placebo for 3 months, from day 1 (day of ablation procedure)	Pulmonary vein isolation	Not reported	15 months
Egami et al. (33)	Randomized controlled study	62	30	32	0.5 mg/day for 2 weeks from the next day after catheter ablation	Pulmonary vein isolation	AF lasting > 30 sec	2 weeks
Imazio et al. (2)	A multicenter, double-blind, randomized trial	336	169	167	Placebo/colchicine 1.0 mg twice daily starting on postoperative day 3 followed by 0.5 mg twice daily for 1 month in patients ≥ 70 kg, halved doses for patients<70 kg or intolerant to the highest dose	CABG, valve replacement, Aorta surgery or combined surgeries	AF lasting 5 min	1 month
Imazio et al. (25)	Double-blind, placebo-controlled, randomized clinical trial	360	180	180	0.5 mg twice daily in patients ≥ 70 kg or 0.5 mg once daily in patients < 70 kg starting between 48 and 72 h before surgery and continued for 1 month after surgery	CABG, valve replacement, Aorta surgery or combined surgeries	AF lasting for more than 30 s	3 months
Sarzaeem et al. (22)	Double-blind, parallel clinical trial	216	108	108	1.0 mg the night before surgery and on the morning of surgery. followed by 0.5 mg twice a day for five days after surgery.	CABG	AF for at least 10 min	6 months
Shvartz et al. (29)	Double-blind, randomized, placebo-controlled clinical trial	101	50	51	1 mg of colchicine 24 h before the surgery, as well as on days 2, 3, 4, and 5 in the postoperative period;	CABG and/or aortic valve replacement	At least 5 min	7days
Tabbalat et al. (26)	Multicentre prospective randomized open label study	360	179	181	2mg 12-24 h prior to surgery and 1 mg 4 h before or immediately after surgery and then 0.5 mg twice daily until hospital discharge. Half the dose was given to patients weighing < 70 kg or intolerant to the full dose.	CABG, valve replacement, Aorta surgery or combined surgeries	at least 5 min	Until hospital discharge
Tabbalat et al. (31)	Prospective, randomized, double-blind, placebo-controlled study	152	81	71	1-mg dose of colchicine or placebo orally 12 to 24 h before surgery followed by a daily dose of 0.5mg until hospital discharge	CABG ?valve replacement orcombined surgeries	at least 5 min	Until hospital discharge
Hua et al. (32)	Randomized controlled study	80	40	40	A 3-month course of colchicine 0.5 mg twice daily or placebo.	Pulmonary vein isolation	AF lasting > 30 sec	3 months
Zarpelon et al. (27)	Prospective, randomized, open, single-center clinical assay	140	71	69	1 mg, twice daily, preoperatively, and of 0.5 mg, twice daily, until hospital discharge. 1 mg was administered to those admitted 12 h or less before surgery.	Elective myocardial revascularization surgery	At least 5 min	Until hospital discharge

AF, atrial fibrillation, CABG, coronary artery bypass grafting.

Table 2 summarizes baseline clinical characteristics of included RCTs. The mean age was 64-years and on average, males accounted for about 72% of subjects. Comparison between colchicine and placebo groups showed no difference in prevalence of hypertension (62.6% vs. 63.7%; *p* = 0.61), diabetes mellitus (31.9% vs. 31.8%; *p* = 0.98), congestive heart failure(12.9% vs. 13.8%; *p* = 0.65), coronary artery disease(32.7% vs. 35.4%; *p* = 0.36), prior myocardial infarction(26.0% vs. 26.5%; *p* = 0.87), chronic obstructive pulmonary disease(11.4% vs. 13.0%; *p* = 0.47), smoking status(31.6% vs. 34.4%; *p* = 0.24).

**TABLE 2:** Overall baseline characteristics of pooled groups of colchicine and placebo patients.

	Colchicine (*n* = 1141)	Placebo (*n* = 1133)	p
Age, yrs	63.99 ± 11.35 (734)	64.73 ± 9.93 (711)	0.19
Male	690/963 (71.7%)	675/943 (71.6%)	0.97
Hypertension	603/963 (62.6%)	601/943 (63.7%)	0.61
Diabetes mellitus	307/963 (31.9%)	300/943 (31.8%)	0.98
Smoking	251/794 (31.6%)	267/776 (34.4%)	0.24
CAD	171/523 (32.7%)	181/511 (35.4%)	0.36
Prior MI	99/381 (26.0%)	96/362 (26.5%)	0.87
COPD	51/448 (11.4%)	57/439 (13.0%)	0.47
CHF	75/581 (12.9%)	79/572 (13.8%)	0.65

Values are n/N (%) or mean ± SD. CAD, coronary artery disease, CHF, congestive heart failure, COPD, chronic obstructive pulmonary disease, MI, myocardial infarction. The descriptive statistics in this table are based on available data in the included studies, as some studies did not reported all patients’ characteristics.

### Quality assessment

**FIGURE 2:**
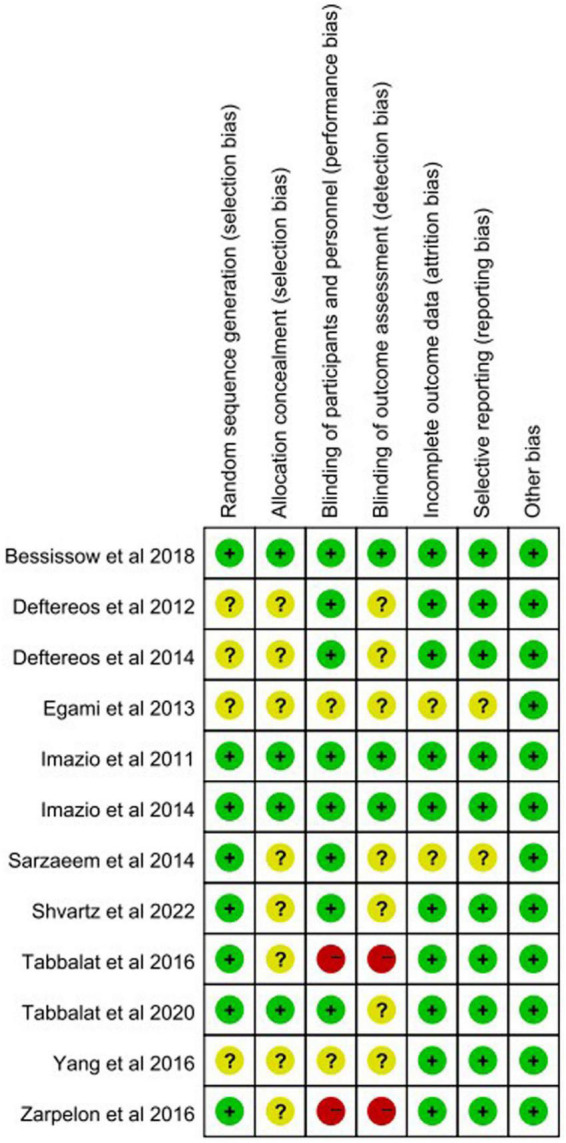
Quality assessments for included studies. Green/plus indicates low risk of bias, yellow/question mark suggests the uncertain risk of bias, and red/minus shows potential high risk of bias.

**FIGURE 3:**
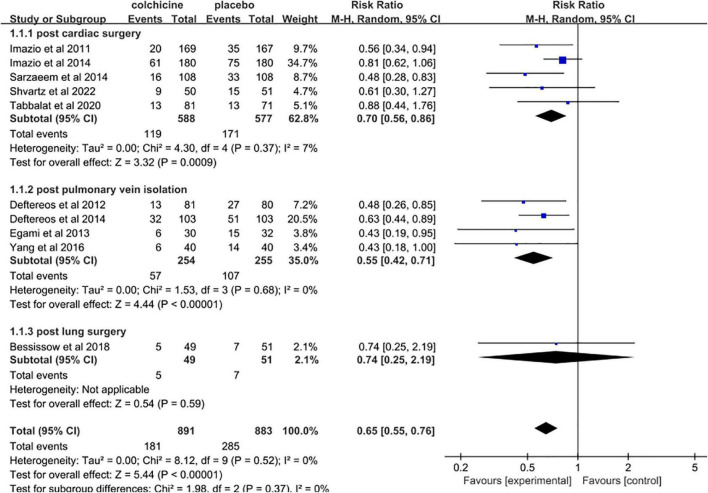
Forest plot indicates risk ratios of post-operative atrial fibrillation with colchicine after the two trials were excluded.

Figure 2 shows the risk of bias assessment of the included studies. We used random sequence generation, allocation concealment, blinding of participants and personnel, blinding of outcome assessment, incomplete outcome data, and selective reporting as the main determinants of the quality of a study. Two trials present high risk of bias in “blinding of participants” and in “blinding outcome assessment” (26, 27). No appreciable difference was found in the efficacy of colchicine for prevention of post-operative AF after the two trials were excluded (Figure 3).

### Prevention of post-operative atrial fibrillation

**FIGURE 4:**
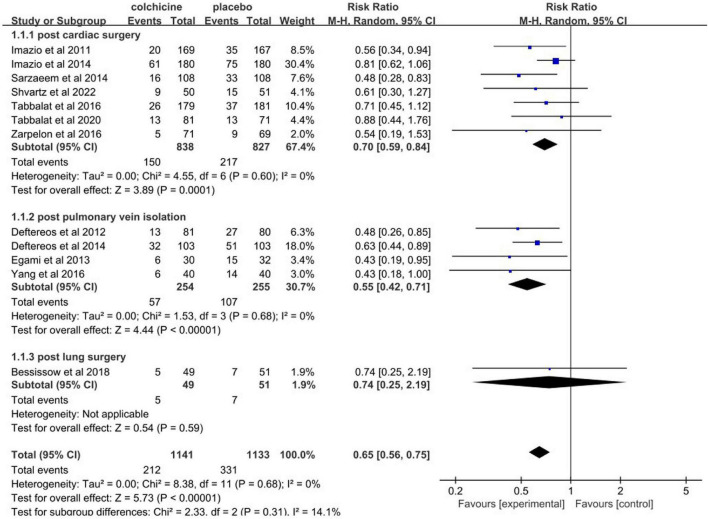
Forest plot indicates risk ratios of post-operative atrial fibrillation with colchicine.

**FIGURE 5:**
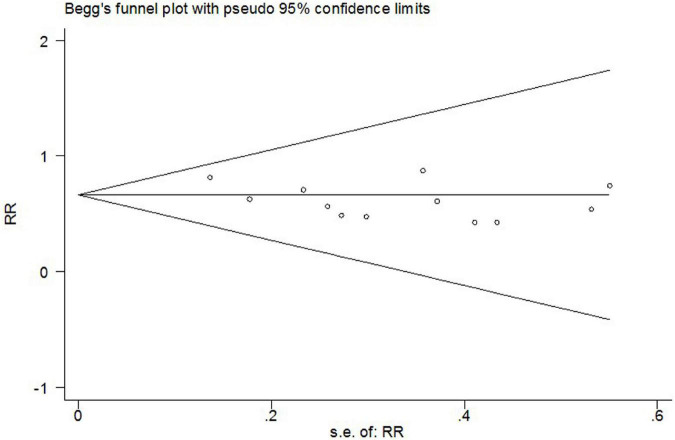
Begg’s test funnel plots to assess the evidence of publication bias.

**FIGURE 6:**
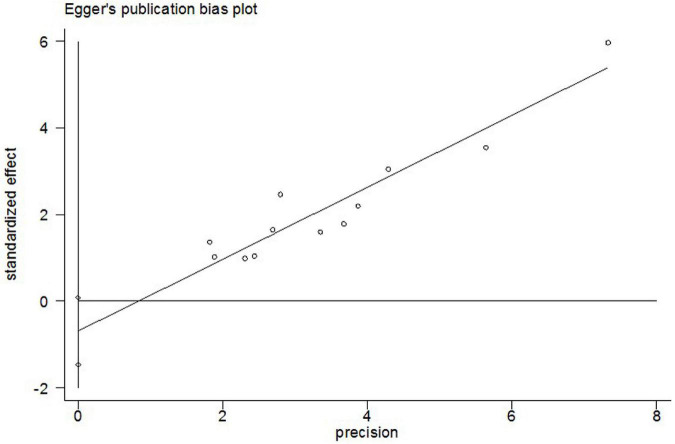
Egger’s test funnel plots to assess the evidence of publication bias.

The incidence of post-operative AF in control groups ranges from 13 to 50%. Colchicine therapy to prevent post-operative AF was associated with a reduction of 35% (RR 0.65, 95% CI 0.56 to 0.75, *p* < 0.001, I^2^ = 0%) when compared to placebo (Figure 4). In the colchicine group, 18.6%(212/1141) suffered from post-operative AF versus 29.2% (331/1133) of control patients. Because of the treatment regime and follow up heterogeneity of the included studies, we used the random-effects model in this meta-analysis. There was low heterogeneity among the included studies using the random method (*I*^2^ = 0%, χ^2^ = 8.38, df = 11, *p* = 0.68). Seven studies showed no differences between colchicine therapy and placebo or usual care. The Begg’s test and Egger’s test were applied to assess publication bias (Figures 5, 6). They show a nearly symmetrical distribution of the plot, which could suggest no significant publication bias.

As displayed in Figure 4, subgroup analysis demonstrates reductions in the occurrence of post-operative AF in post cardiac surgery (RR 0.70, 95% CI 0.59 to 0.84, *p* < 0.001, *I*^2^ = 0%) and in post pulmonary vein isolation (RR 0.55, 95% CI 0.42 to 0.71, *p*<0.001, *I*^2^ = 0%) when comparing to placebo. Whereas in post lung surgery, colchicine therapy did not statistically significantly reduce the incidence of post-operative AF (RR 0.74, 95% CI 0.25 to 2.19, *p* = 0.59).

### Sensitivity analysis

**FIGURE 7:**
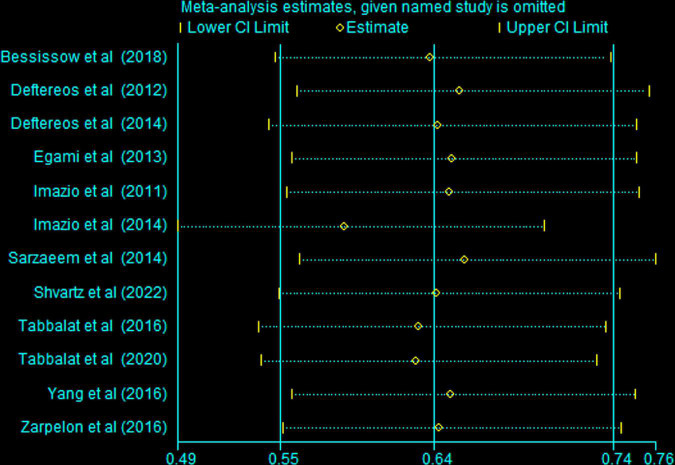
Sensitivity analysis by removing each study in turn.

In this meta-analysis, we conducted sensitivity analysis by removing each study in turn. As showed in Figure 7, the overall risk ratio ranged from 0.59 (95%CI 0.49 to 0.70) (Imazio et al.) to 0.65 (95%CI 0.56 to 0.76) (Sarzaeem et al.). The 95% CIs and the point estimates did not change greatly. Therefore, our results are not dependent upon a single study.

### Adverse effects related to colchicine therapy

**FIGURE 8:**
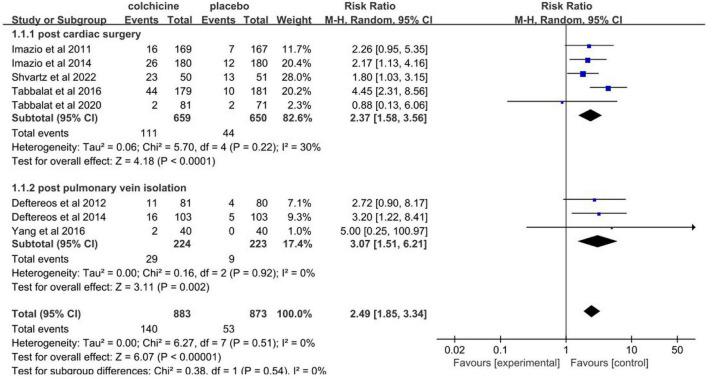
Forest plot shows gastrointestinal adverse effects.

**FIGURE 9:**
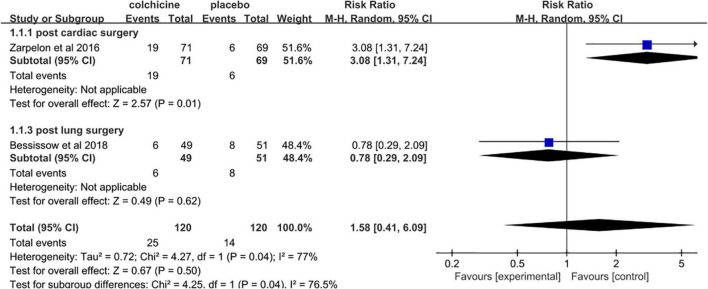
Overall risk of infection.

**FIGURE 10:**
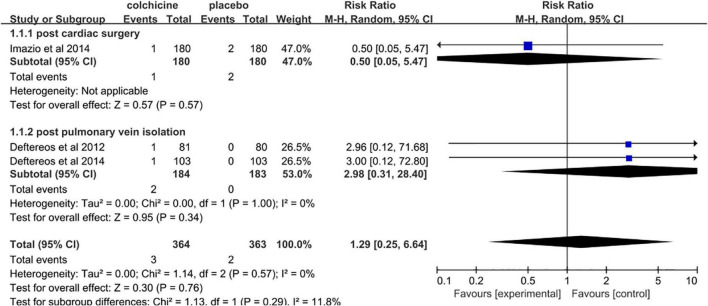
Overall risk of hepatotoxicity.

Pooled data analysis revealed that colchicine therapy was related to a 2.49 fold increased risk for gastrointestinal adverse effects compared to usual care or placebo (RR = 2.49, 95% CI 1.85 to 3.34, *p* < 0.001, *I*^2^ = 0%). 15.9% (140/883) who received colchicine therapy experienced gastrointestinal adverse effects versus 6.1% (53/873) of control patients (Figure 8). Only two trials reported the incidence of infection. The overall incidence of infection was about 20.8% (25/120) for colchicine and 11.7% (14/120) for controls (RR = 1.58, 95% CI 0.41 to 6.09, *p* = 0.50, *I*^2^ = 77%) (Figure 9). Three trials reported the incidence of hepatotoxicity. The overall incidence of hepatotoxicity was about 0.8% (3/364) for colchicine and 0.6% (2/363) for controls (RR = 1.29, 95% CI 0.25 to 6.64, *p* = 0.76, *I*^2^ = 0%) (Figure 10).

### Major adverse events

**FIGURE 11:**
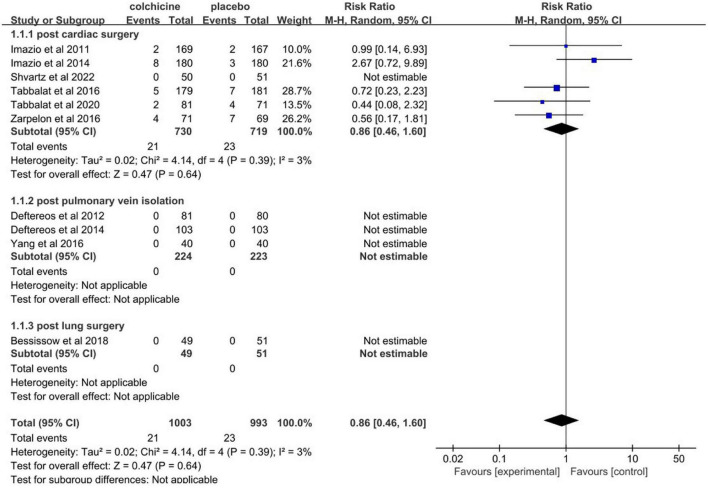
Forest plot for overall risk of major adverse effects (all-cause death and stroke).

Ten trials [Bessissow et al. (30); Deftereos et al. (23); Deftereos et al. (14); Imazio et al. (2); Imazio et al. (25); Shvartz et al. (29); Tabbalat et al. (26); Tabbalat et al. (31); Hua et al. (32) and Zarpelon et al. (27)] reported major adverse events (stroke and all-cause death). Pooled data revealed that the overall incidence of major adverse events was about 2.1% (21/1003) for colchicine and 2.3% (23/993) for controls (RR = 0.86, 95% CI 0.46 to 1.60, *p* = 0.64, *I*^2^ = 3%) (Figure 11).

Accordingly, there was no evidence to reject the null hypothesis of no difference between colchicine therapy and placebo or usual care in major adverse events.

### Early treatment discontinuation

**FIGURE 12:**
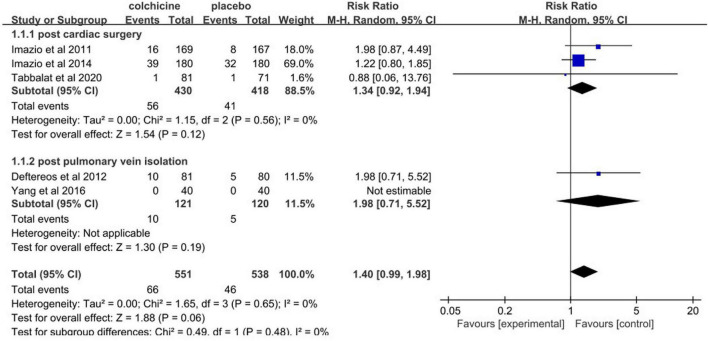
Forest plot for overall risk of early treatment discontinuation.

Early treatment discontinuation was reported in five studies. Pooled data revealed that there was no significant difference in the rate of early treatment discontinuation between colchicine group and placebo group (RR: 1.40; 95% CI: 0.99 to 1.98; *p* = 0.06) (Figure 12).

### Hospital length-of-stay

**FIGURE 13:**
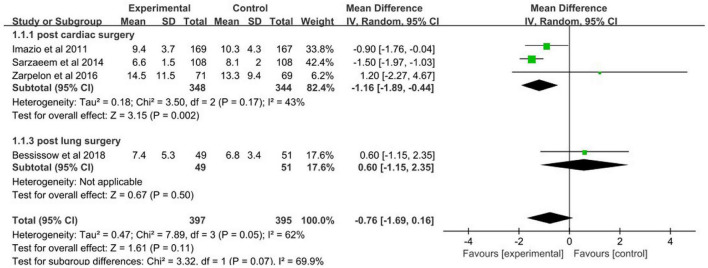
Forest plot for hospital length of stay.

Colchicine treatment was not statistically significantly related to a reduction in hospital length-of-stay (mean difference: –0.76 days, 95% CI: –1.69 to 0.16; *p* = 0.07) (Figure 13). But there was a trend that patients in colchicine treatment group had a shorter hospital length-of-stay (*p* = 0.07).

## Discussion

To the best of our knowledge, this meta-analysis is the largest one on the efficacy of colchicine to prevent post-operative AF. This meta-analysis included 12 RCTs which enrolled a total of 2274 patients undergoing cardiac surgery, lung surgery and PVI for AF. It demonstrates that the administration of colchicine in the perioperative period results in significantly lower rate of post-operative AF. However, colchicine was also associated with a higher rate of occurrence of side effects, especially gastrointestinal adverse effects, compared to placebo. Previous meta-analyses have shown inconsistent results regarding the efficacy of colchicine for prevention of post-operative AF. Only three trials were included in the studies of Lee et al. and Wang et al. respectively (24, 28). Our studies provide more information regarding the adverse effects related to colchicine therapy, and major adverse events (35).

The exact pathophysiology of post-operative AF is remained to elucidate. It is regarded as multifactorial, including autonomic nervous system imbalance, pericardial inflammation, hypoxia, and metabolic and electrolyte disturbances (10–12, 36). Mounting evidence suggests that inflammation is a major factor in the development of post-operative AF (12, 36). Inflammatory biomarkers such as C-reactive protein and interleukin-6 are associated with the incidence of post-operative AF (13–15, 37, 38). The NLRP3 inflammasome activation plays a vital role in the secretion of the inflammatory cytokines which are involved in the occurrence of AF (39). The administration of corticosteroids is related to the reduction of AF recurrences after cardiac surgery or PVI (12, 40). Microtubules play a key role in cellular cytoskeletal and intracellular transport activities. Colchicine can disrupt microtubule assembly in cells (41, 42). Moreover, colchicine inhibits the assembly of the NLRP3 inflammasome, prevents the activation of caspase-1, and reduces the release of IL-1β, IL-6 and superoxide (43–45). Both the whole-cell and single channel currents activated by cell mechanical deformations were completely blocked by colchicine administration in rat atrial fibroblasts (46). Together, colchicine has the ability to inhibit leukocytes functions and exert its anti-inflammatory function (19, 21). Colchicine also has anti-fibrosis effects and a variety of effects on endothelial function (47).Post-operative AF is related to an increased hospital stay, poorer neurocognitive outcomes, elevated health care costs, and increased incidence of stroke and death (2, 6, 7). Our meta-analysis reveals that there was a trend that patients in colchicine treatment group had a shorter hospital length-of-stay when comparing to patients in placebo treatment group. Although colchicine therapy increased the occurrence of gastrointestinal adverse effects, the rate of early treatment discontinuation did not rise in colchicine treatment group. Furthermore, pooled data suggested colchicine treatment did not increase the major adverse events. There was no significant difference in the incidence of hepatotoxicity and infection between the two groups. However, only a few trials reported the incidence of hepatotoxicity and infection, and more trials are needed. Given the undesirable clinical outcomes, related health care costs, and safety of colchicine, colchicine may be recommended as a prophylactic drug in patients to reduce the incidence of post-operative AF.

Previous studies show that many drugs such as amiodarone, beta-blockers, colchicine, corticosteroids, magnesium, and statins, have been assessed for potential roles to lower the incidence of post-operative AF (2, 16–18, 48–50). However, only amiodarone and beta-blockers get a class I recommendation by international guideline (1). Amiodarone is the most promising agent for prevention of post-operative AF. Yet, the side effects of amiodarone, specifically pulmonary toxicity, limit its routine use in clinical settings. In a large RCT, perioperative metoprolol in non-cardiac surgery was related to an increased risk of death and stroke (51). Our study demonstrates that colchicine is a useful and safe agent for prevention of post-operative AF. Although in post lung surgery subgroup, colchicine therapy did not reduce the incidence of post-operative AF. There was just one study with only 100 patients included in this subgroup. The study was underpowered to evaluate efficacy outcomes. The results were promising as other studies showed similar point estimates in post cardiac surgery and post pulmonary vein isolation subgroup. Further large studies are required to investigate the efficacy of colchicine in patients undergoing lung surgery. Our study also shows a higher incidence of gastrointestinal adverse effects in colchicine group. Nonetheless, compared to control group, the rate of early treatment discontinuation was not significant in colchicine group. However, instead of routine prophylactic use of colchicine, it is suggested to consider colchicine in special patients at higher risk of post-operative AF, such as those with chronic obstructive pulmonary disease, valvular heart disease, obesity, advanced age, left atrial enlargement, and heart failure (52). Another way to reduce the incidence of gastrointestinal adverse effects is to optimize dose and duration of colchicine therapy. However, the optimal dose and duration of colchicine are still confusing. In this meta-analysis, the dose and duration of colchicine in the included studies were highly variable, from 0.5mg once a day until hospital discharge to 0.5 mg twice daily for three months. Further studies are required to evaluate the ideal dose and duration of colchicine in prevention of post-operative AF.

### Study limitations

Our meta-analysis has some potential limitations. One limitation is the relative heterogeneity in the type of operations (cardiac surgery, lung surgery, and interventional procedure) performed among the included studies. Some operations are related to a higher incidence of AF when compared to others. However, the underlying mechanisms that lead to AF are similar. Another potential limitation is various colchicine administration protocols and follow-up time periods. One potential confounding factor is AF under detection during the follow-up time. Different studies included had different means of AF detection and follow-up protocols. Finally, the use of perioperative antiarrhythmic medications is not the same for all the studies included in this meta-analysis.

## Conclusion

The results of our meta-analysis suggest that colchicine therapy could lower the incidence of post-operative AF, and could be considered as a complementary prophylaxis. Further studies are needed to determine the optimal colchicine treatment regime to minimize the incidence of adverse events.

## Data availability statement

The original contributions presented in this study are included in the article/supplementary material, further inquiries can be directed to the corresponding author.

## Ethics statement

The studies involving human participants were reviewed and approved by the included studies have passed ethical requirements. The patients/participants provided their written informed consent to participate in this study.

## Author contributions

PG, YF, and QS have made substantial contributions to conception and design of the study, searched literature, and extracted data from the collected literature. MJ, LG, and CM analyzed the data. SZ and JZ wrote the manuscript. ZZ and JH revised the manuscript. All authors read and approved the final manuscript.
